# Rare variants of *RNF213* and moyamoya/non-moyamoya intracranial artery stenosis/occlusion disease risk: a meta-analysis and systematic review

**DOI:** 10.1186/s12199-017-0680-1

**Published:** 2017-11-02

**Authors:** Xin Liao, Jing Deng, Wenjie Dai, Tong Zhang, Junxia Yan

**Affiliations:** 1 Department of Epidemiology and Health Statistics, XiangYa School of Public Health, Central South University, Shang Mayuanling, KaiFu District, Changsha, 410078 China; 2 Department of Neurology, the Second Hospital of Hebei Medical University, Heping West Road, Xinhua District, Shijiazhuang, 050000 China

**Keywords:** Moyamoya disease, Intracranial stenosis disease, *RNF213*, rare variants, Genetics, Review

## Abstract

**Background:**

The p.R4810K and other rare variants of ring finger protein 213 gene (*RNF213*) were illustrated as susceptibility variants for moyamoya (MMD) and non-moyamoya intracranial artery stenosis/occlusion disease (ICASO) recently. However, the effect sizes of p.R4810K were in great discrepancy even in studies of the same ethnic population and firm conclusions of other rare variants have been elusive given the small sample sizes and lack of replication. Thus, we performed this study to quantitatively evaluate whether or to what extent the rare variants of *RNF213* contribute to MMD and ICASO in different populations.

**Methods:**

A systematic search of PubMed, EMBASE, ISI web of science, CNKI, and WANFANG DATA was conducted up to 5 September 2017. Pooled odds ratios (ORs) with 95% confidence intervals (CIs) were calculated using random- or fixed-effect models based on the between-study heterogeneity. The subgroup analyses were performed by the ethnicity and family history. Sensitivity and publication bias analysis were performed to test the robustness of associations. All the statistical analyses were conduct using STATA 12.0.

**Results:**

Twenty studies including 2353 MMD cases and 5488 controls and 11 studies including 1778 ICASO cases and 3140 controls were included in this study. Pooled ORs indicated that *RNF213* p.R4810K significantly increased MMD and ICASO risk in East Asians with great effect sizes of discrepancy (dominant model: odds ratios 184.04, 109.77, and 31.53 and 10.07, 28.52, and 5.59 for MMD and ICASO, respectively, in Japan, Korea, and China). It significantly increased familial MMD risk in Japan, Korea, and China with 5 ~ 36 times larger effect sizes than that for sporadic ones in each country (dominant model ORs 1802.44, 512.42, 1109.02 and 134.35, 99.82, and 30.52, respectively, for familial and sporadic cases). The effect sizes of *RNF213* p.R4810K to sporadic MMD were 3 ~ 4 times larger in Japan and Korea than those in China. *RNF213* p.R4810K also increased the ICASO risk in Japan and Korea with 2 ~ 4 times larger effect sizes than that in China (dominant model ORs 10.71, 28.52, and 5.59, respectively). Another two rare variants- p.E4950D and p.A5021V significantly increased MMD risk in Chinese population (dominant model ORs 9.06 and 5.01, respectively). Various other rare variants in *RNF213* were identified in Japanese, Chinese, European, and Hispanic American populations without association evidence available yet.

**Conclusions:**

This meta-analysis shows the critical roles of *RNF213* p.R4810K in MMD especially familial MMD and ICASO in Japan, Korea, and China. Except for *RNF213* p.R4810K, MMD seems to have more complex determiners in China. Distinct genetic background exists and other environmental or genetic factor(s) may contribute to MMD. Studies focused on delineating the ethnicity-specific factors and pathological role of *RNF213* variants in MMD and ICASO are needed.

**Electronic supplementary material:**

The online version of this article (10.1186/s12199-017-0680-1) contains supplementary material, which is available to authorized users.

## Background

Moyamoya disease (MMD) is an idiopathic steno-occlusive disease of intracranial arteries characterized by progressive bilateral and occasionally unilateral stenosis and occlusion of the distal internal carotid artery, with frequent involvement of the middle cerebral artery and anterior cerebral artery, and by the abnormal development of a hazy network of basal collateral vessels [1, 2]. MMD occurs worldwide, but its prevalence is highest in East Asian countries, including Japan, Korea, and China [3–6]. There are two incidence peaks for MMD, one in children around 10 years of age and another in adults in their 30–40 years [7]. Affected individuals are at risk for intracranial hemorrhagic or ischemic stroke, seizures, cognitive impairment, and developmental delays [1]. Although much progress has been made in our understanding of MMD, the etiology is still not well understood, and no medication can inhibit or reverse its progression. At present, direct or indirect neurosurgical revascularization is the mainstay MMD treatment [8]. Pathological clues for early diagnosis and novel therapeutic approaches are needed.

Based on the existence of familial cases and the observation of a strong ethnicity effect of MMD, a genetic contribution is strongly suspected [9, 10]. In 2011, two research groups identified ring finger protein 213 gene (*RNF213*) on 17q25.3 as a novel susceptibility gene for MMD in Japan and East Asian population, respectively [11, 12]. The *RNF213* rare variant p.R4810K [rs112735431, corresponding to c.14429G>A on the basis of the NCBI Reference sequence NM_001256071.2, theminor allele frequency (MAF) in the 1000 Genome is 0.0012] significantly increases MMD risk in Japan, Korea, and China (odds ratios (ORs) were 338.94, 135.63, and 14.70 in a dominant model, respectively) [12]. Further replication studies confirmed that *RNF213* p.R4810K was a founder mutation in East Asian and absent from European, Hispanic, and African-descent MMD cases [13–20]. Recently, several studies further revealed that *RNF213* p.R4810K was associated with intracranial artery stenosis/occlusion that did not meet the diagnostic criteria for MMD (ICASO) in Japan, Korea, and China [21–25]. They hypothesized that some cases of ICASO ascribed to unknown etiology or atherosclerosis might be an early onset MMD which was misdiagnosed by the traditional imaging diagnostic methods [21–23, 25]. Since the therapeutic strategies are different for these diseases, genetic testing or sequencing of *RNF213* is proposed for MMD and ICASO diagnosis [21–23, 25]. However, the carrier rates of *RNF213* p.R4810K in MMD and ICASO were greatly discrepant in different studies. It varied from 66.7 to 90.1% in Japanese and Korean MMD patients, and to a lesser degree in Chinese ones with a range from 9.4 to 31.4%, the effect sizes were significantly different even in studies of the same ethnic population [12, 13, 18, 19, 26, 27]. For ICASO, there were more than 20% of patients who carried *RNF213* p.R4810K in Japan and Korea, while the rates were much lower in China [21–25]. The lack of consistency of these studies is probably due to population stratification or small sample sizes in individual studies with inadequate statistical power. In addition, many non-p.R4810K rare variants (MAF < 0.005 in 1000 Genome database) in *RNF213* have been identified in both Asian and Caucasian MMD cases recently [11–17, 19, 28]. However, *RNF213* is a large gene (encodes 5207 amino acids) and harbors a number of missense variants in the general population as well as the patients [29]. The false assignment of pathogenicity may lead to incorrect therapeutic or prognostic assessments of patients [30]. Thus, scientifically quantitative evaluation of the contributions of *RNF213* rare variants to MMD and ICASO is urgently needed for the future applications and studies.

Previously, Sun et al. performed meta-analysis to investigate the associations between *RNF213* variants (mainly p.R4810K) and MMD susceptibility with eight studies included [31]. They concluded that *RNF213* p.R4810K is closely associated with MMD risk. Recently, some other studies were subsequently performed. Considering the discrepant results and only MMD was involved in the previous meta-analysis, we performed this study to quantitatively evaluate whether or to what extent the rare variants of *RNF213* contribute to MMD and ICASO in different populations.

## Methods

This meta-analysis was conducted according to the Human Genome Epidemiology Network guidelines and followed the published recommendations to improve the quality of meta-analyses of genetic association studies [32]. We assessed the quality of reporting of genotyping on the basis of the Strengthening the Reporting of Genetic Association Studies statement [33].

### Literature search strategy

Electronic databases PubMed, EMBASE, Web of Science, WANFANG DATA, and China National Knowledge Infrastructure (CNKI) were used to retrieve potentially relevant articles on human genetic studies of MMD and ICASO that had been published up to 5 September 2017. Search terms used were RNF213*[tw] or RNF 213*[tw] or ring finger protein 213*[tw]. Articles in all languages were searched and translated as necessary. After relevant articles were retrieved, references were also checked for other potentially relevant articles not found in the initial search.

### Selection criteria and data extraction

We included related studies evaluating associations of *RNF213* rare variants with proven MMD or ICASO (using computed tomography angiography or magnetic resonance angiography or digital subtraction angiography) in all ethnicities. The detailed inclusion criteria were (1) well-designed case control studies to investigate the relationship between at least one genetic variant of *RNF213* and MMD or ICASO, or case-only studies which investigated the carrier rate of *RNF213* variants in MMD or ICASO and the carrier rates of the target variants were available in the general population; (2) clear diagnostic criteria of MMD and ICASO; (3) original papers contained independent and sufficient genotype data to calculate ORs and 95% confidence intervals (CIs); (4) all variants included in the meta-analysis should be evaluated in at least two published studies. Where duplicate or overlapped datasets existed, only the largest study was included. The studies without essential information or with overlapped data, review articles, case reports, and animal models were excluded. For the variants identified just in one study or the sample number that was limited to perform association analysis, a qualitative systematic review was performed.

Data were extracted by two of the authors (XL and JD), and differences were resolved by consensus (JY). For each included study, the following information was extracted: first author, year of publication, study population (country), mean age, familial history of MMD, numbers of patients and controls, frequency of genotypes, and Hardy–Weinberg equilibrium (HWE) status. Where genotype frequencies for each variant were unavailable, we estimated the number of cases per genotype category by using published information on risk allele frequencies and ORs for MMD or ICASO. The HWE of controls was obtained either directly from the article or indirectly by calculating from genotype distributions using a web-based program (http://www.oege.org/software/hwe-mr-calc.shtml). Quality assessment of primary studies was performed using Newcastle–Ottawa quality assessment scale (NOS) [34]. Each study with NOS scores ≥ 6 was regarded as a high-quality study.

### Statistical analysis

Statistical analyses were conducted using STATA12.0 software (Stata Corporation, College Station, TX, USA). Frequency of the genotypes and alleles between MMD/ICASO group and control group were compared using Chi-square or Fisher exact test. For each genetic variant with more than one publication, meta-analysis was performed to determine a pooled OR and 95% CI according to dominant, recessive and allelic models by using a fixed- or random-effect model. The significance of the pooled OR was determined using *Z* test, and *p* < 0.05 was considered statistically significant.

Heterogeneity among studies was assessed using Cochran *Q* test and quantified by using Higgins *I* ^2^ statistic. CIs for *I* ^2^ were also calculated. For *Q* test, *p* < 0.05 was considered as having significant heterogeneity. For variant association showing significant inter-study heterogeneity (*Q* test, *p* values < 0.05, and *I* ^2^ > 50%), the random-effect model was used as the pooling method; otherwise, the fixed-effect model was used. To evaluate ethnic-specific effects, subgroup analysis was performed according to the nationality of the study population. Publication bias was assessed by using the Egger regression asymmetry test and visualization of funnel plots if more than seven studies were included, and the significance was set at the *p* < 0.05 level. Sensitivity analysis was performed by sequentially excluding individual study to calculate the pooled OR of the remaining studies and assess the stability of the results.

## Results

### Main characteristics of all the available studies

**Fig. 1:**
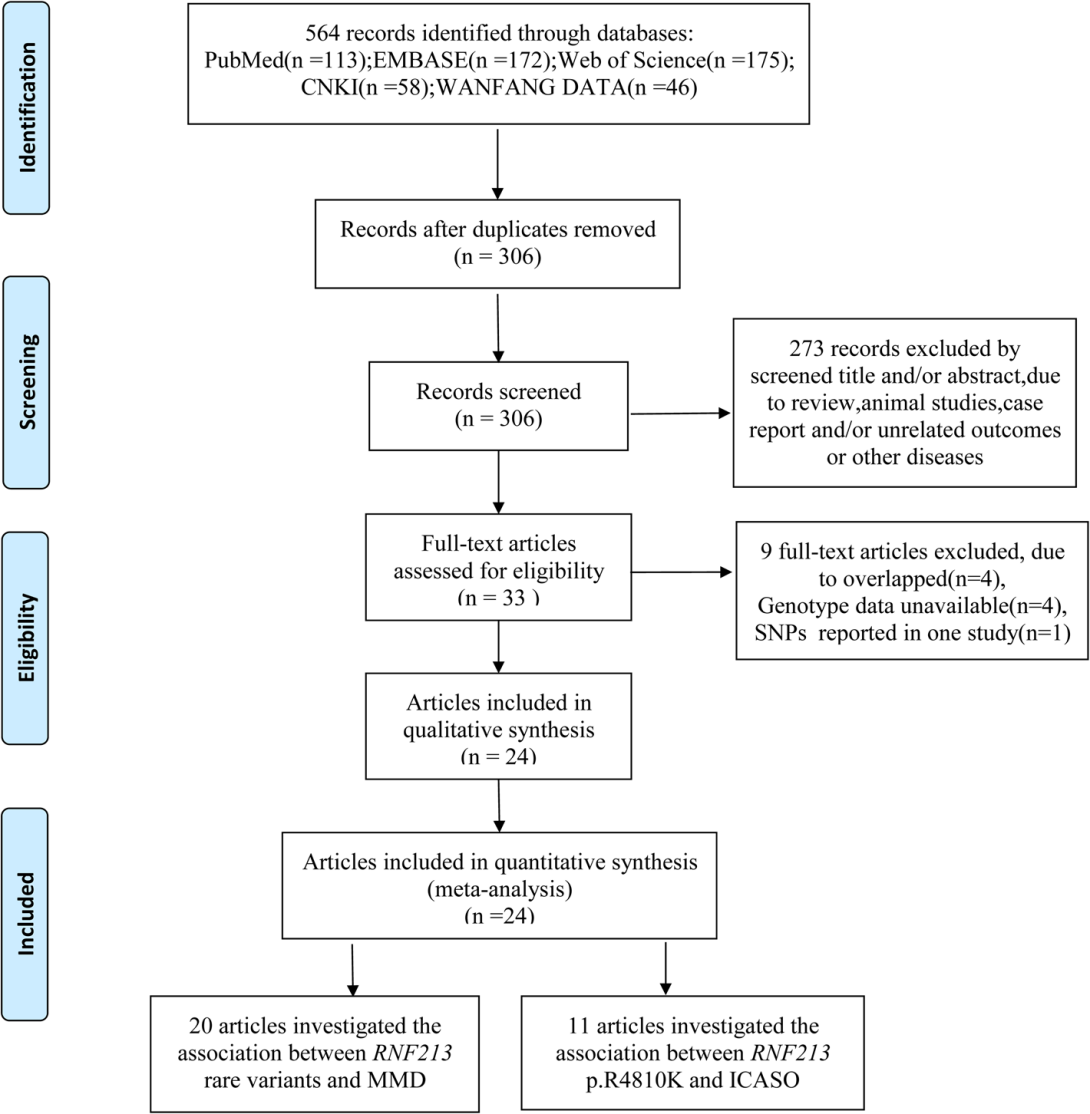
PRISMA flow diagram of study selection process

Five hundred sixty-four articles were identified through the database check, and no article was identified through the related references check. After screening for duplication and eligibility, data from 24 studies met the inclusion criteria and was included. A detailed workflow chart showing the study selection is presented in Fig. 1.

In total, twenty articles investigated the association between seven *RNF213* rare variants (p.R2092C, p.D4013N, p.R4062Q, p.A4399T, p.R4810K, p.E4950D, and p.A5021V) and MMD [11–19, 21–24, 27, 35–40]; eleven articles investigated the association between *RNF213* p.R4810K and ICASO [21–25, 36, 38, 40–43]. These studies encompassed mainly Japanese, Korean, and Chinese populations. Detailed characteristics of all eligible studies are shown in Table 1.

**Table 1:** Detailed characteristics of all eligible studies for the association with *RNF213* rare variants and MMD or ICASO

Diseases	Rare variants	Author and reference	Year	Country	Sample size		Mean age (years)		Family history of MMD (%)		Genotype^a^		Carrier rate (%)		OR(95% CI)^b^	NOS	HWE
					Case	Control	Case	Control	Case	Control	Case	Control	Case	Control			
MMD	p.R4810K (rs112735431) G>A	Kamada et al. [11]	2011	Japan	105	457	–	–	–	–	17/84/4	438/19/0	83.81	4.16	119.33 (59.66–238.7)	8	0.65
		Liu et al. [12]	2011	Japan	161	384	29.9 ± 21.4	61.8 ± 10.2	–	–	16/135/10	374/9/1	90.06	2.60	338.94 (150.32–764.20)	7	0.01
		Liu et al. [12]	2011	Korea	38	223	38.7 ± 14.1	40.7 ± 10.9	–	–	8/30/0	217/6/0	78.95	2.69	135.63 (44.02–417.86)	8	0.84
		Liu et al. [12]	2011	China	52	150	22.7 ± 17.9	39.4 ± 10.9	–	–	40/11/1	148/2/0	23.08	1.33	22.20 (4.77–103.26)	7	0.93
		Miyatake et al. [35]	2012	Japan	204	283	22.7 ± 17.9		20.1	–	36/153/15	278/5/0	82.35	1.77	259.47 (99.86–674.15)	7	0.88
		Miyawaki et al. [21]	2012	Japan	48	25	48.4 ± 18.7	49.8 ± 16.1	–	–	7/40/1	25/0/0	85.42	0.00	282.20 (15.45–5153.83)^c^	7	–
		Wu et al. [13]	2012	China	170	507	35.8 ± 13.2	37.2 ± 16.9	2.9	0	148/21/1	505/2/0	12.94	0.39	37.53 (8.72–161.47)	9	0.96
		Wang et al. [27]	2013	China	96	96	43.0 ± 13.7	42.6 ± 9.4	–	–	87/8/1	95/1/0	9.38	1.04	9.83 (1.22–79.17)	9	0.96
		Miyawaki et al. [22]	2013	Japan	30	110	46.7 ± 18.4	39.1 ± 14.1	–	–	10/19/1	108/2/0	66.67	1.8182	108.00 (21.99–530.34)	7	0.92
		Bang et al. [23]	2015	Korea	131	51	51.3 ± 13.7^d^	–	–	–	32/98/1	51/0/0	75.57	0.00	315.34 (18.92–5254.74)^c^	7	–
		Lee et al. [15]	2015	China	36	500	–	–	22.2	–	30/6/0	498/2/0	16.67	0.40	49.80 (9.64–257.29)	7	0.96
		Moteki et al. [16]	2015	Japan	103	95	27[9–42]^e^	–	26.2	–	27/71/5	93/2/0	73.79	2.11	130.89 (44.02–417.86)	7	0.92
		Huang et al. [36]	2015	China	52	80	41.6 ± 11.2	40 ± 10.4	–	–	45/6/1	79/1/0	13.46	1.25	12.29 (1.46–103.10)	8	0.96
		Kim et al. [37]	2016	Korea	165	294	21.3 ± 13.6	40.9 ± 10.9	27.9	–	40/112/13	286/8/0	75.76	2.72	111.72 (50.82–245.58)	7	0.81
		Shoemaker et al. [17]	2016	Japan	5	5	–	–	–	–	2/3/0	5/0/0	60.00	0.00	18.20 (0.67–494.80)^c^	7	–
		Shoemaker et al. [17]	2016	Korea	11	11	–	–	–	–	3/8/0	11/0/0	72.73	0.00	55.86 (2.53–1231.23)^c^	7	–
		Shoemaker et al. [17]	2016	China	6	6	–	–	–	–	5/0/1	6/0/0	16.67	0.00	3.55 (0.12–105.82)^c^	7	–
		Huang et al. [18]	2016	China	81	100	42.7 ± 12.2	40.2 ± 11.9	–	–	69/10/2	98/2/0	14.81	2.00	8.52 (1.85–39.29)	8	0.92
		Bang et al. [24]	2016	Korea	288	83	45.9 ± 12.7	56.0 ± 12.2	10.1	–	89/199/0	82/1/0	69.10	1.20	183.35 (25.12–1338.15)	7	0.96
		Zhang et al. [19]	2016	China	255	300	26.7 ± 14.7	28.0 ± 15.9	13.7	–	175/78/2	300/0/0	31.37	0.00	275.67 (16.99–4473.13)^c^	9	–
		Park et al. [38]	2017	Korea	25	100	49 ± 7.1	–	–	–	7/18/0	98/2/0	72.00	2.00	126.00 (24.20–656.00)	9	0.92
		Jang et al. [39]	2017	Korea	264	1516	44.4				86/177/1	1479/37/0	67.42	2.44	87.38 (57.75–132.22)	7	0.63
		Shinya et al. [40]	2017	Japan	5	100	46.4 ± 19.3	68.8 ± 15.8	–	–	1/4/0	98/2/0	80.00	2.00	196.00 (14.55–2639.78)	9	0.92
	p.R2092C (rs139265462) C>T	Shoemaker et al. [17]	2015	European	74	74	–	–	–	–	73/1/0	74/0/0	1.35	0.00	3.04 (0.12–75.86)^c^	7	–
		Shoemaker et al. [17]	2015	Hispanic	6	6	–	–	–	–	5/1/0	6/0/0	16.67	0.00	3.55 (0.12–105.82)^c^	7	–
	p.D4013N (rs397514563) G>A	Liu et al. [12]	2011	Czech	8	120	–	–	–	–	7/1/0	120/0/0	12.50	0.00	48.20 (1.81–1286.73)^c^	7	–
		Cecchi et al. [14]	2014	European	22	12	–	–	–	–	19/3/0	12/0/0	13.64	0.00	4.49 (0.21–94.47)^c^	6	–
		Zhang et al. [19]	2016	China	255	300	26.7 ± 14.7	28.0 ± 15.9	13.7	–	254/1/0	300/0/0	0.39	0.00	3.54 (0.14–87.33)^c^	9	–
	p.R4062Q G>A	Liu et al. [12]	2011	German	42	164	–	–	–	–	41/1/0	164/0/0	2.38	0.00	11.89 (0.48–297.23)^c^	7	–
		Moteki et al. [16]	2015	Japan	370	279	–	–	–	–	369/1/0	279/0/0	0.27	0.00	2.27 (0.09–55.92)^c^	7	–
		Zhang et al. [19]	2016	China	255	300	26.7 ± 14.7	28.0 ± 15.9	13.7	–	254/1/0	300/0/0	0.39	0.00	3.54 (0.14–87.33)^c^	9	–
	p.A4399T (rs148731719) G>A	Kamada et al. [11]	2011	Japan	63	53	–	–	–	–	59/4/0	51/2/0	6.35	3.77	1.73 (0.30–9.83)	8	0.89
		Miyatake et al. [35]	2012	Japan	204	188	22.7 ± 17.9	–	20.1	–	191/12/1	172/16/0	6.37	8.51	0.73 (0.34–1.56)	7	0.54
		Wu et al. [13]	2012	China	170	507	48.4 ± 18.7	49.8 ± 16.1	–	–	142/27/1	462/45/0	16.47	8.88	2.60 (1.53–4.43)	9	0.3
		Wang et al. [27]	2013	China	96	96	43.0 ± 13.7	42.6 ± 9.4	–	–	96/0/0	85/11/0	0.00	11.46	0.04 (0.00–0.66)^c^	9	0.55
		Huang et al. [36]	2015	China	52	80	41.6 ± 11.2	40 ± 10.4	–	–	49/3/0	80/0/0	5.77	0.00	11.38 (0.58–225.08)^c^	8	–
	p.E4950D (rs371441113) G>C	Liu et al. [12]	2011	China	52	150	22.7 ± 17.9	39.4 ± 10.9	–	–	50/2/0	150/0/0	3.85	0.00	14.90 (0.70–315.60)^c^	7	–
		Wu et al. [13]	2012	China	170	507	48.4 ± 18.7	49.8 ± 16.1	–	–	169/1/0	507/0/0	0.59	0.00	8.98 (0.36–221.54)^c^	9	–
		Zhang et al. [19]	2016	China	255	300	26.7 ± 14.7	28.0 ± 15.9	13.7	–	253/2/0	300/0/0	0.78	0.00	5.93 (0.28–124.02)^c^	9	–
	p.A5021V (rs138130613) C>T	Liu et al. [12]	2011	China	52	150	22.7 ± 17.9	39.4 ± 10.9	–	–	50/2/0	150/0/0	3.85	0.00	14.90 (0.70–315.60)^c^	7	–
		Wu et al. [13]	2012	China	170	507	48.4 ± 18.7	49.8 ± 16.1	–	–	169/1/0	507/0/0	0.59	0.00	8.98 (0.36–221.54)^c^	9	–
		Wang et al. [27]	2013	China	50	90	–	–	–	–	47/3/0	89/1/0	6.00	1.11	2.39 (0.39–14.80)	9	0.91
		Huang et al. [36]	2015	China	52	80	41.6 ± 11.2	40 ± 10.4	–	–	49/3/0	78/2/0	5.77	2.50	5.68 (1.57–15.98)	8	0.96
ICASO	p.R4810K (rs112735431) G>A	Miyawaki et al. [21]	2012	Japan	41	25	62.3 ± 11.3	49.8 ± 16.1	–	–	32/8/1	25/0/0	21.95	0.00	14.91 (0.83–268.43)^c^	7	–
		Miyawaki et al. [22]	2013	Japan	84	110	61.5 ± 12.6	39.1 ± 14.1	–	–	64/20/0	108/2/0	23.81	1.82	16.88 (3.82–74.58)	7	0.92
		Bang et al. [23]	2015	Korea	221	51	–	–	–	–	144/77/0	51/0/0	34.84	0.00	55.24 (3.36–907.41)^c^	7	–
		Huang et al. [36]	2015	China	64	80	42.5 ± 12.2	40 ± 10.4	–	–	58/5/1	79/1/0	9.38	1.25	8.17 (0.96–69.74)	8	0.96
		Shang et al. [41]	2015	China	139	300	–	–	–	–	138/1/0	299/1/0	0.72	0.33	2.17 (0.43–10.63)	8	0.98
		Bang et al. [24]	2016	Korea	234	83	56.0 ± 12.2	–	–	–	184/50/0	82/1/0	21.37	1.20	22.28 (3.03–164.07)	7	0.96
		Kim et al. [25]	2016	Korea	31	1516^f^	–	–	–	–	17/140	1479/37/0	45.16	2.44	32.92 (15.11–71.74)	7	0.63
		Park et al. [38]	2017	Korea	31	100	49 ± 14.1	–	–	–	24/7/0	98/2/0	22.58	2.00	14.58 (2.85–74.69)	9	0.92
		Zhang et al. [42]	2017	China	715	507	–	–	–	–	709/6/0	505/2/0	0.84	0.39	2.14 (0.43–10.63)	7	0.96
		Xue et al. [43]	2017	China	114	268					106/8/0	267/1/0	7.54	0.37	20.15 (2.49–163.08)	9	0.98
		Shinya et al. [40]	2017	Japan	104	100	–	68.80 ± 15.8	–	–	94/10/0	98/2/0	9.62	2.00	5.21 (1.11–24.42)	9	0.92

*MMD* moyamoya disease, *ICASO* non-moyamoya intracranial artery stenosis/occlusion disease, − not available

^a^Genotype presented as wild type/heterozygous/homozygous

^b^OR(95% CI) was calculated in the dominant model

^c^We applied a half-integer continuity correction to all four cells if the event rates were zero

^d^Mean age of 352 intracranial stenosis patients(including MMD and ICASO) in this study. ^e^Median age at onset and interquartile range ^f^For this case-only study, we use 1516 general Korean individuals reported by Jang M.A et al. as control [39]

### Quantitative synthesis and heterogeneity analysis

#### *RNF213* rare variants and MMD

##### Association between *RNF213* p.R4810K and MMD

The most robust variant associated with MMD was *RNF213* p.R4810K. Nineteen articles representing 23 studies evaluated their associations, of which 8 were conducted in Japanese; 7, in Korean; and 8, in Chinese with a total of 2331 MMD cases and 5476 controls.

**Fig. 2:**
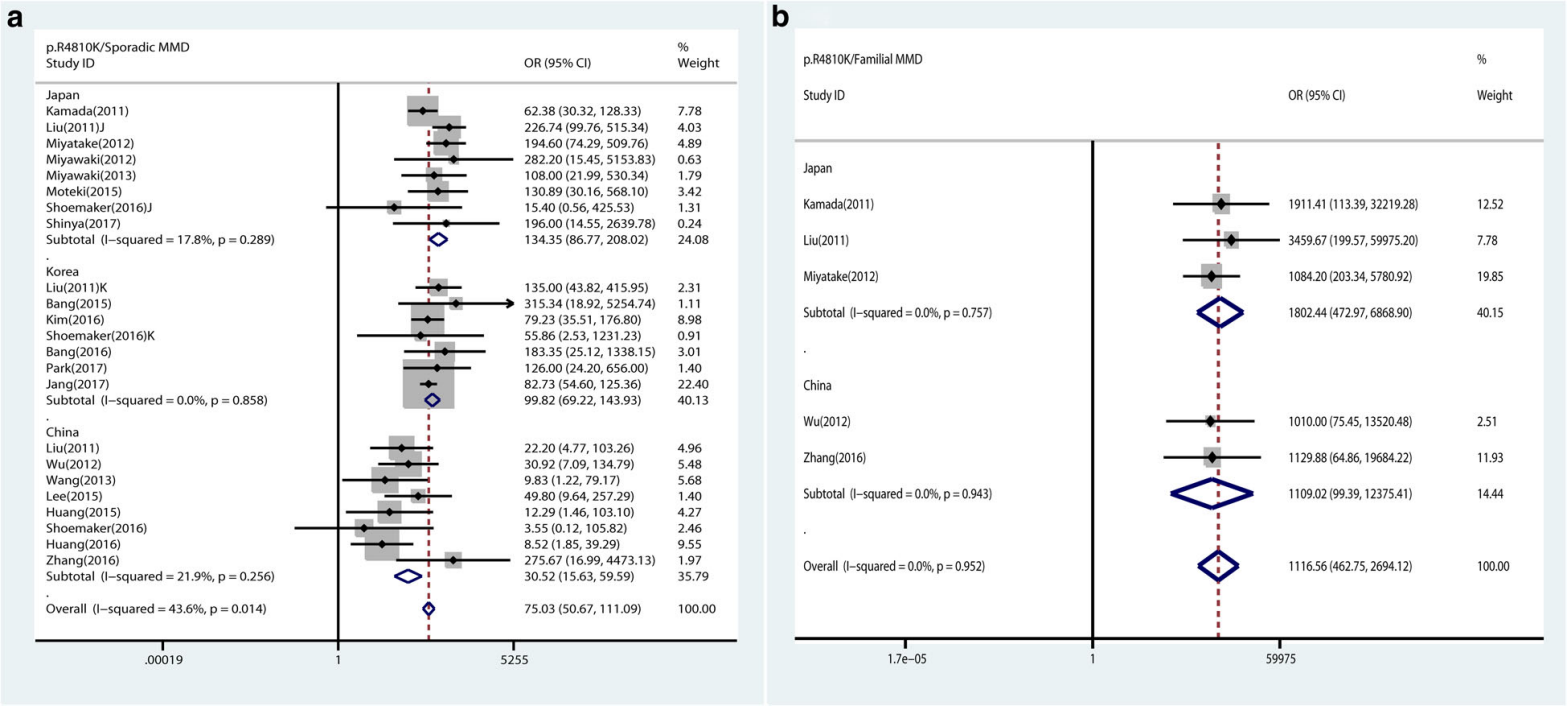
Forest plots for the association of *RNF213* p.R4810K with MMD under the dominant model. **a** Forest plots of *RNF213* p.R4810K and sporadic MMD in different subgroup populations under the dominant model. **b** Forest plots of *RNF213* p.R4810K and familial MMD in different subgroup populations under the dominant model

The pooled results suggested a significant association between p.R4810K and MMD in all genetic models (dominant model: OR 85.91, 95% CI 56.36–130.95, *p* < 0.0001) (Table 2). Country-based subgroup analysis showed that p.R4810K robustly associated with MMD in Japanese, Korean, and Chinese populations with 3.5 ~ 5.8 times effect sizes difference (dominant model ORs 184.04, 109.77, and 31.53 in Japan, Korea, and China, respectively) (Table 2, Fig. 2a).

**Table 2:** Main results of the pooled ORs in meta-analysis for the association between *RNF213* rare variants and MMD or ICASO

Variants	*N*	Sample size (case/control)	Dominant model				Recessive model				Allelic model			
			OR (95% CI)	*I* ^2^ (%)	*p* ^b^	*p* ^c^	OR (95% CI)	*I* ^2^ (%)	*p* ^b^	*p* ^c^	OR (95% CI)	*I* ^2^ (%)	*p* ^b^	*p* ^c^
MMD														
(1) p.R4810K (rs112735431)														
Total	23	2331/5476	85.91 (56.36–130.95)^a^	51.6	0.002	< 0.0001	13.19 (6.37–27.31)	0.0	0.886	< 0.0001	46.54 (36.73–58.97)	0.0	0.497	< 0.0001
Subgroup analysis														
Country														
Japan	8	661/1459	184.04 (119.56–283.29)	0.0	0.453	< 0.0001	19.52 (6.30–60.47)	0.0	0.666	< 0.0001	58.64 (40.88–84.12)	0.0	0.621	< 0.0001
Familial	3	131/1124	1802.44 (472.97–6868,90)	0.0	0.757	< 0.0001	51.70 (11.53–231.80)	0.0	0.676	< 0.0001	77.40 (50.44–117.66)	0.0	0.370	< 0.0001
Sporadic	8	530/1459	134.35 (86.77–208.02)	17.8	0.289	< 0.0001	9.93 (2.96–33.30)	0.0	0.884	< 0.0001	52.08 (35.41–76.60)	0.0	0.504	< 0.0001
Korea	7	922/2278	109.77 (76.30–157.93)	0.0	0.889	< 0.0001	17.38 (3.08–98.07)	38.6	0.196	0.001	42.81 (30.25–60.57)	0.0	0.804	< 0.0001
Familial	1	46/294	512.42 (130.85–2006.64)	–	–	< 0.0001	33.09 (1.56–700.58)	–	–	0.025	69.41 (30.92–155.83)	–	–	< 0.0001
Sporadic	7	876/2278	99.82 (69.22–143.93)	0.0	0.858	< 0.0001	17.62 (3.14–99.02)	42.4	0.176	0.001	49.52 (35.11–69.85)	0.0	0.933	< 0.0001
China	8	748/1739	31.53 (16.18–61.46)	24.2	0.236	< 0.0001	5.48 (1.64–18.35)	0.0	0.999	0.006	31.51 (16.02–62.00)	22.0	0.3	< 0.0001
Familial	2	40/807	1109.02 (99.39–12,375.41)	0.0	0.943	< 0.0001	338.33 (12.08–9475.51)	–	–	0.001	575.09 (60.07–5505.59)	0.0	0.9891	< 0.0001
Sporadic	8	743/1739	30.52 (15.63–59.59)	21.9	0.256	< 0.0001	5.12 (1.40–18.77)	0.0	0.998	0.014	29.77 (15.19–58.35)	5.3	0.390	< 0.0001
Total														
Familial	6	217/2225	1116.56 (462.75–2684.12)	0.0	0.849	< 0.0001	51.86 (14.18–189.64)	0.0	0.720	< 0.0001	85.35 (59.09–123.27)	22.7	0.156	< 0.0001
Sporadic	23	2139/5476	75.03 (50.67–111.09)^a^	43.6	0.014	< 0.0001	9.41 (4.36–20.32)	0.0	0.957	< 0.0001	46.34 (36.32–59.13)	0.0	0.633	< 0.0001
(2) p.R2092C (rs139265462)														
Total	2	80/80	3.27 (0.32–33.80)	0.0	0.949	0.321	–	–	–	–	3.13 (0.31–31.28)	0.0	0.974	0.331
(3) p.D4013N (rs397514563)														
Total	3	285/432	6.47 (0.96–43.55)	0.0	0.443	0.055	–	–	–	–	6.18 (0.92–41.33)	0.0	0.433	0.06
(4) p.R4062Q														
Total	3	400/559	4.64 (0.72–29.96)	0.0	0.798	0.107	–	–	–	–	4.62 (0.72–29.76)	0.0	0.798	0.107
(5) p.A4399T (rs148731719)														
Total	5	585/924	1.15(0.41–3.25)^a^	70.8	0.008	0.79	4.80 (0.49–47.02)	0.0	0.611	0.178	1.19 (0.45–3.19)^a^	69.2	0.011	0.727
Subgroup analysis														
Japan	2	267/241	0.85(0.43–1.68)	0.0	0.374	0.636	2.78 (0.11–68.63)	–	–	0.532	0.90 (0.46–1.76)	0.0	0.426	0.767
China	3	318/683	1.13(0.06–21.30)^a^	80.1	0.007	0.933	8.98 (0.36–221.54)	–	–	0.179	1.04 (0.07–15.72)^a^	79.6	0.007	0.975
(6) p.E4950D (rs371441113)														
Total	3	477/957	9.06 (1.49–55.27)	0.0	0.915	0.017	–	–	–	–	9.00 (1.48–54.78)	0.0	0.917	0.017
(7) p.A5021V (rs138130613)														
Total	4	324/827	5.01(1.57–15.98)	0.0	0.738	0.006	–	–	–	–	4.91 (1.55–15.53)	0.0	0.733	0.007
ICASO														
p.R4810K (rs112735431)														
Total	11	1778/3140	13.89 (8.01–24.09)	37.0	0.140	< 0.0001	2.70 (0.28–26.38)	0.0	0.764	0.394	13.01 (7.55–22.42)	18.3	0.269	< 0.0001
Subgroup analysis														
Japan	3	229/235	10.71 (3.97–28.91)	0.0	0.537	< 0.0001	1.89 (0.07–48.17)	–	–	0.7	10.00 (3.74–26.77)	0.0	0.570	< 0.0001
Korea	4	517/1750	28.52 (11.04–73.67)	0.0	0.779	< 0.0001	–	–	–	–	24.16 (9.71–60.13)	0.0	0.845	< 0.0001
China	4	1032/1155	5.59 (2.12–14.75)	11.6	0.335	0.001	3.80 (0.15–94.95)	–	–	0.416	5.76 (7.55–22.42)	13.0	0.328	< 0.0001

*N* number of studies, − not available

^a^ORs were calculated under random-effects model

^b^ *p* value for *Q* test

^c^ *p* value for *Z* test, *I* ^2^, Higgins *I* ^2^ statistic

Further stratified analysis by family history of MMD (familial index cases or sporadic cases) in each ethnic population revealed that *RNF213* p.R4810K significantly increased familial MMD risk in Japanese, Korean, and Chinese population, with 5 ~ 36 times larger effect sizes than that in sporadic cases (Table 2). For the sporadic MMD, the effect sizes of *RNF213* p.R4810K were in great discrepancy in different countries. It was 3 ~ 4 times larger in Japanese and Korean than that in Chinese (dominant model ORs 134.35, 99.82, and 30.52, respectively) (Table 2, Fig. 2a, b).

##### Association between *RNF213* non-p.R4810K variants and MMD

Except *RNF213* p.R4810K, the associations between the other six rare variants (p.R2092C, p.D4013N, p.R4062Q, p.A4399T, p.E4950D, and p.A5021V) and MMD were evaluated in at least two published studies. The detailed information was presented in Tables 1 and 2.

**Fig. 3:**
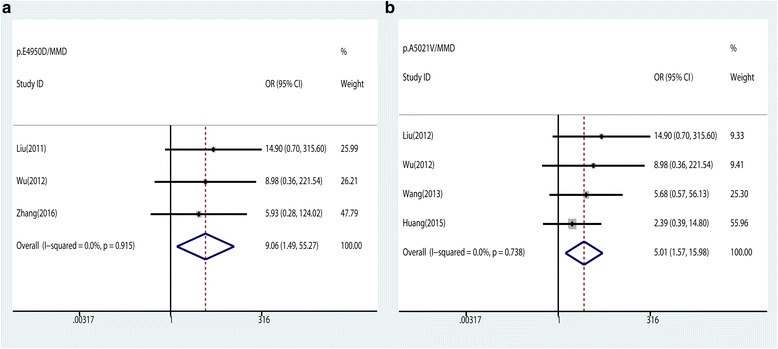
Forest plots for the association of *RNF213* p.E4950D and p.A5021V with MMD under the dominant model. **a** Forest plots of *RNF213* p.E4950D and MMD in Chinese population under the dominant model. **b** Forest plots of *RNF213* p.A5021V and MMD in Chinese population under the dominant model

There were two rare variants—p.E4950D and p.A5021V—significantly associated with MMD in Chinese population in the pooled analysis (pooled ORs 9.06 and 5.01, 95% CIs 1.49–55.27 and 1.57–15.98, respectively, in a dominant model) (Fig. 3a, b). No significant associations were observed between the other four variants and the susceptibility of MMD in this meta-analysis (Table 2).

##### Association between *RNF213* p.R4810K and ICASO

**Fig. 4:**
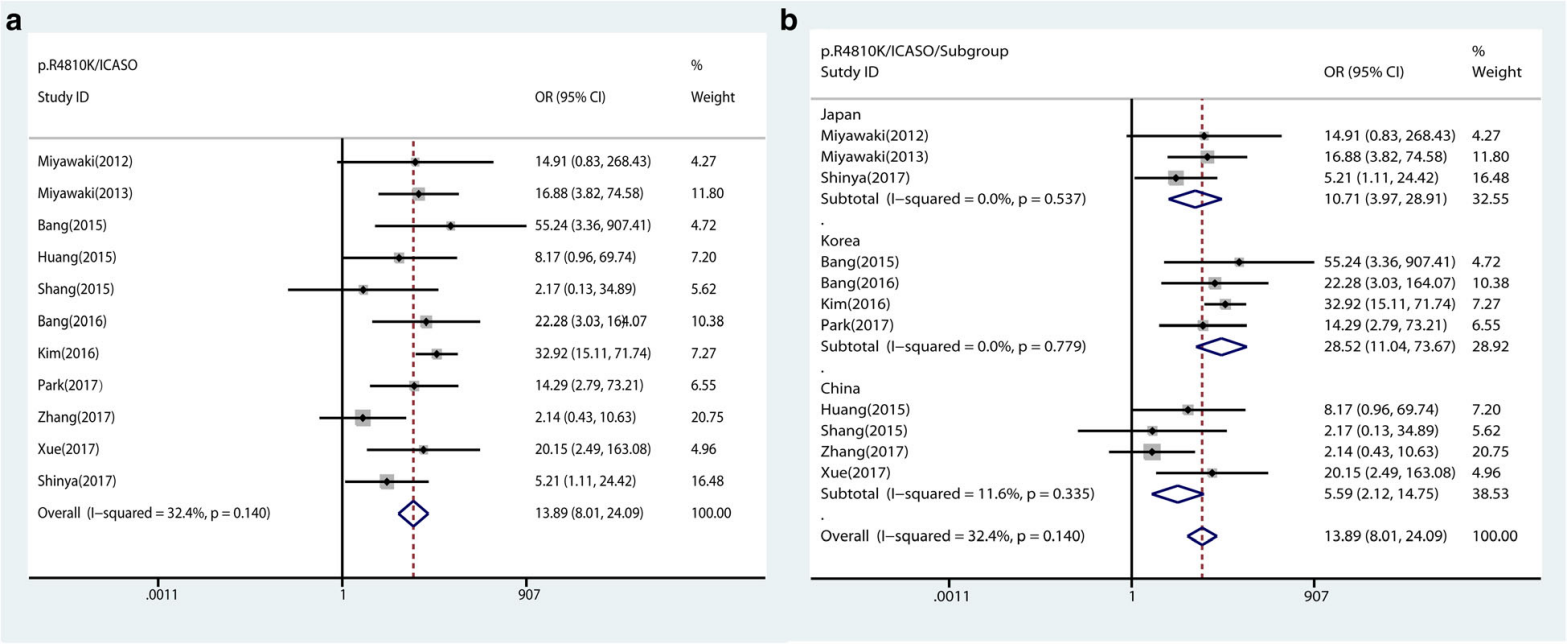
Forest plots for the association of *RNF213* p.R4810K with ICASO under the dominant model. **a** Forest plots of *RNF213* p.R4810K and ICASO in the general population under the dominant model. **b** Forest plots of *RNF213* p.R4810K and ICASO in the subgroup populations under the dominant model

The association between p.R4810K and ICASO was investigated by 11 studies, including 1778 ICASO patients and 3140 controls. Result showed that p.R4810K was significantly associated with the risk of ICASO (dominant model: OR 13.89, 95% CI 8.01–24.09, *p* < 0.0001 (Table 2 and Fig. 4a).

Subgroup analysis showed that the strongest association was observed in Korea (dominant model: OR 28.52; 95% CI 11.04–73.67, *p* < 0.0001), followed by that in Japan (dominant model: OR 10.71, 95% CI 3.97–28.91, *p* < 0.0001) and China (dominant model: OR 5.59, 95% CI 2.12–14.75, *p* = 0.001) (Table 2, Fig. 4b).

### Publication bias

**Fig. 5:**
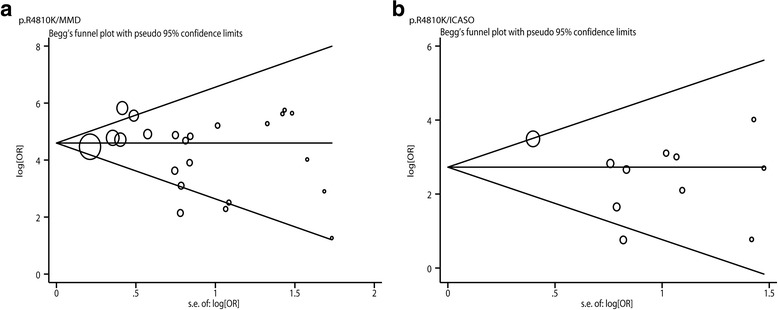
Funnel plots for the association of *RNF213* p.R4810K with MMD and ICASO under the dominant model. **a** Funnel plot of *RNF213* p.R4810K and MMD under the dominant model. **b** Funnel plot of *RNF213* p.R4810K and ICASO under the dominant model

Owning to the association between *RNF213* p.R4810K and MMD that was investigated by 23 studies, we used Begg’s funnel plot and the Egger regression asymmetry test to assess the publication bias of these studies. In the dominant model, the results of Begg’s funnel plot (continuity corrected *p* value, 0.561) and the Egger regression asymmetry test (*t* = − 1.27, *p* = 0.218) did not find significant asymmetry (Fig. 5a). For the association between *RNF213* p.R4810K and ICASO, no significant publication bias was observed (Fig. 5b).

### Sensitivity analysis

Sensitivity analysis was performed by sequentially excluding individual study for each meta-analysis to assess the stability of the results. For the association between *RNF213* p.R4810K and MMD or ICASO, corresponding pooled ORs showed no significant change when sequentially excluded one study from each meta-analysis, which indicated that these results are stable and reliable (Additional file 1: Figure S1).

### Systematic review of other *RNF213* rare variants and MMD

**Fig. 6:**
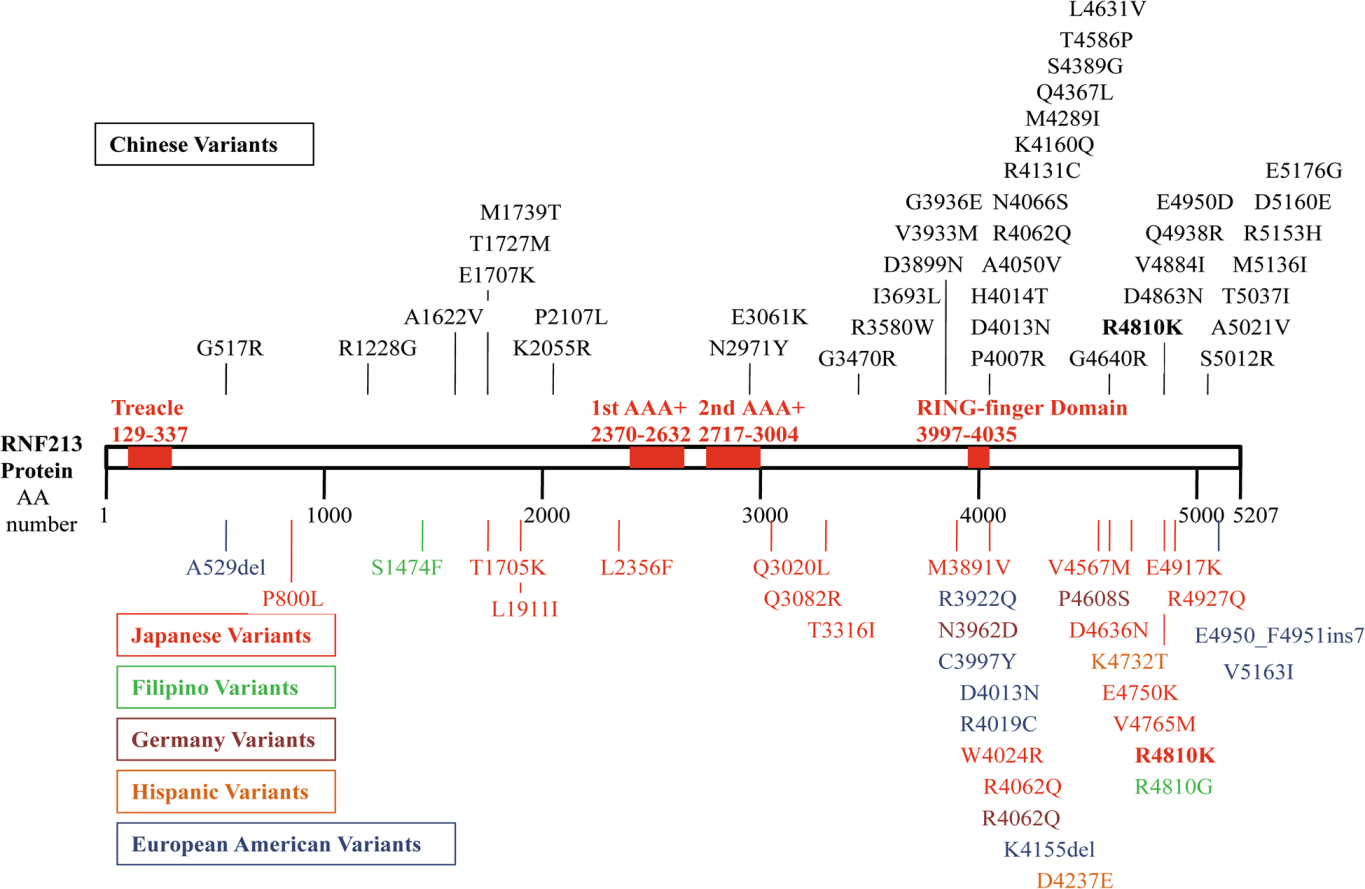
Other rare variants of *RNF213* identified in MMD individuals. The figure was adapted from Cecchi et al. [14] and Koizumi et al. [29]. Variants identified in different populations are marked in different colors respectively. AA, amino acid; AAA+, ATPase associated with diverse cellular activities domain. The bold-styled variant mean the most robust one associated with MMD

Except variants mentioned above, various other rare variants of *RNF213* were identified in Japanese, Chinese, European, and Hispanic American populations (Fig. 6 and Additional file 2: Table S1) [11–14, 16, 17, 28].These variants were not found in control subjects and were detected in only one patient, suggesting that they had potential causative effects in MMD development.

## Discussion

MMD is a rare idiopathic intracranial vascular disorder with strong genetic components. Genetic study of familial MMD clearly indicated autosomal dominant inheritance pattern [44]. *RNF213* was the first identified susceptibility gene for MMD recently. We performed this study to quantitatively evaluate whether or to what extent the rare variants of *RNF213* contribute to MMD and ICASO in different populations. The main results showed that *RNF213* p.R4810K significantly increased familial MMD risk in Japanese, Korean, and Chinese population (dominant model ORs 1802.44, 512.42, and 1109.02), with 5 ~ 36 times larger effect sizes than that for sporadic cases (dominant model ORs 134.35, 99.82, and 30.52) (Table 2). The pooled results were similar to the original report by Liu et al. [12] and illustrated that genetic screening of *RNF213* p.R4810K in Japanese, Korean, and Chinese population especially in the people with familial history of MMD would be an effective approach to identify asymptomatic patients [44]. For the sporadic cases, significant effect sizes difference was observed in different countries. The effect sizes of *RNF213* p.R4810K were 3 ~ 4 times greater in Japanese and Korean population than that in Chinese. This illustrated that distinct genetic background may exist and other environmental or genetic factor(s) may contribute to sporadic MMD. In this study, we found another two rare variants—p.E4950D and p.A5021V—in *RNF213* significantly increased MMD risk in Chinese population in the pooled analysis (pooled ORs 9.06 and 5.01, 95% CIs 1.49–55.27 and 1.57–15.98, respectively, in the dominant model). In addition, more than 40 other rare missense variants of *RNF213* were identified in Chinese MMD cases but absent in controls (such as p.D4013N, p.R4062Q, p.D4863N, p.D5160E, and p.E5176G) [12, 13, 19]. Of them, p.D4013N and p.R4062Q have been independently reported by different studies, highly indicating the causative effects [12–14, 19]. Recently, Kobayashi et al. found that *RNF213* p.D4013N-transfected human umbilical vein endothelial cells displayed significant lowered migration activity which was similar with the experiment result of p.R4810K transfection and strongly indicated the disease-causing effect [28]. However, due to the low allele frequency and the limited sample size, it was difficult to get association evidences for them. Furthermore, except for the rare variants mentioned above, more than half of Chinese MMD has not been identified the possible disease-causing variants of *RNF213* [19]. MMD appears to have more complex determiners in China. In addition, even in Japan and Korea, the majority of carriers with *RNF213* p.R4810K remain unaffected with MMD [26]. Unknown factors are considered to overlay the genetic predisposition to develop MMD [45]. Both genetic and environmental triggers should be explored in the future studies.

Except for the variants mentioned above in the Asian population, various *RNF213* rare variants were identified in MMD cases worldwide [12, 14]. Even *RNF213* p.R4810K was not identified in European, Hispanic, or Black descent MMD patients, other rare variants in *RNF213* were identified in these populations, such as p.A529del, p.R3922Q, p.N3962Q, p.C3997Y, p.D4013N, and p.R4019C (Fig. 6) [12, 14, 20, 28]. Due to the low allele frequency and the limited sample size, no associations were observed between these variants and MMD. However, there is evidence suggesting that many of these variants are disease causing. First, the variants are either not present or present at extremely low frequencies (MAF < 0.001) in the Exome Variant Server database. Second, most of these variants located in the C terminus of *RNF213* protein, which is where the *RNF213* p.R4810K founder variant located [29]. Even with limited information about these variants, causative effect was highly suspected. The genetic heterogeneity may partly explain why manifestations of MMD vary by geographic regions and ethnic groups.

In this study, we also found that *RNF213* p.R4810K was significantly associated with ICASO in Japan and Korea (pooled OR 10.71 and 28.52, 95% CI 3.97–28.91 and 11.04–73.67, respectively) and to a less degree in Chinese population (pooled OR 5.59; 95% CI 2.12–14.75). About the association results, there are two possible explanations: (1) MMD has been misclassified as ICASO due to the atypical manifestation with the absence of one or two diagnostic criteria and lead to the spurious association between *RNF213* p.R4810K and ICASO or (2) *RNF213* p.R4810K is indeed associated with ICASO. Currently, MMD was diagnosis based on the findings of magnetic resonance angiography or digital subtraction angiography: (1) steno-occlusive lesions around the terminal portions of the internal carotid arteries (including proximal portions of the anterior and middle cerebral arteries), (2) moyamoya vessels at the base of the brain appearing as abnormal vascular networks, (3) findings 1 and 2 are present bilaterally [1]. Bang et al. analyzed 352 consecutive ischemic patients within the middle cerebral artery distribution and found that the occurrence of *RNF213* p.R4810K increased with the number of observed angiographic criteria. They demonstrated that the current criteria is limited in distinguishing MMD and ICASO, and a substantial proportion of patients with adult-onset MMD may be misclassified as having ICASO [23]. However, they found that more than one fifth of ICASO patients confirmed by high-resolution magnetic resonance imaging and conventional angiography had *RNF213* p.R4810K variant in a subsequent research, which demonstrated that the *RNF213* p.R4810K is also a high-risk variant for ICASO [24]. We prefer to agree that there exist a new entity of ICASO caused by the *RNF213* p.R4810K variant, which can be differentiated from ICASO caused by atherosclerosis by using genetic analysis [21, 22]. However, similar with MMD, geographic and ethic discrepancies are also highly indicated for ICASO. In China, *RNF213* p.R4810K variant contributed less extent of ICASO risk compared to that in Korea and Japan (pooled ORs were 5.59, 28.52, and 10.71 in China, Korea, and Japan, respectively). Similar with MMD, ethnicity-specific genetic and environmental factors may contribute to this discrepancy. Further well-designed genetic epidemiology studies focusing on ethnicity-specific risk factors such as choosing the relative genetically homogenous population and comprehensively collecting the detailed environmental factors of ICASO are needed.

To date, the mechanisms of how *RNF213* p.R4810K and other rare variants lead to intracranial vascular lesions are still unknown [29]. An in vitro functional study revealed that *RNF213* p.R4810K affected neither the transcription level nor the ubiquitin ligase activity of the protein [12]. Knockdown of *RNF213* in zebrafish leads to abnormal sprouting and irregular diameter of intracranial vessels, suggesting some role of *RNF213* in the vascular formation [12]. Hitomi et al. observed reduced angiogenic activity and genomic instability in endothelial cells derived from induced pluripotent stem cells of p.R4810K-mutated patients [46, 47]. However, ablation of Rnf213 in mice did not induce any apparent abnormality of the vascular system [45, 48]. Unknown factors are considered to overlay the genetic predisposition in the *RNF213* p.R4810K carrier to develop vascular lesions [49]. Recently, Kobayashi et al. found that *RNF213* p.R4810K showed a reduced angiogenesis of transgenic mouse response to hypoxia in vivo [49]. Scholz et al. found that *Rnf213* was a co-regulated gene for the WNT signaling enhancer R-spondin3 (*RSPO3*) and identified that endothelial RSPO3-driven non-canonical WNT/Ca(2+)/NFAT signaling as a critical maintenance pathway of the remodeling vasculature [50]. Banh et al. found that protein-tyrosine phosphatase-1B (*PTP1B*) controlled non-mitochondrial oxygen consumption by regulating *RNF213* to promote tumor survival during hypoxia and concluded that PTP1B/RNF213/α-KGDD pathway was critical for survival of tumors in the hypoxic microenvironment [51] The investigation of WNT signaling and PTP1B/RNF213/α-KGDD pathway in cells expressing *RNF213* R4810K and other rare variants under different environmental condition such as hypoxia and chronic inflammation is expected to provide answers to the pending questions.

The limitations of this study should be considered. First, due to the fact that we analyzed the association between rare variants and diseases, the number of cases and controls involved in the meta-analysis for moderate effect rare variants may be less powered, studies with larger sample size and high quality are needed to explore the associations in the future; second, MMD and ICASO appears to have complex determiners, with both genetic predisposition and environmental triggers. Unknown modifier factor(s) may also be contributory to MMD and ICASO. Multivariate analysis to adjust for the confounding factors such as behavior or clinical or biochemical factors in our meta-analysis was not available. Further comprehensive studies focusing on multiple ethnicity-specific factors are needed; third, ICASO may represent a broad spectrum of diseases and there are various phenotypes (i.e., bilateral M1 occlusion or unilateral M2 stenosis and so on), which may belong to different clinical entities. Due to no more clinical information available in the original papers, subgroup analysis could not be performed according to these factors, which may lead to bias. Further studies with detailed clinical features are needed; fourth, this analysis was constrained to studies which were published and deposited in English and Chinese databases, the other databases were not available, and selection bias could not be excluded (Additional file 3).

## Conclusions

This comprehensive systematic review and meta-analysis reveals that the critical roles of *RNF213* p.R4810K in MMD especially familial MMD and ICASO in Japan, Korea, and China. It significantly increases MMD and ICASO risk in Japanese and Korean population and to a less degree in Chinese population. Except for *RNF213* p.R4810K, another two rare variants—p.E4950D and p.A5021V—increased MMD risk in Chinese population. MMD seems to have more complex determiners in China. Distinct genetic background exists, and other environmental or genetic factor(s) may contribute to MMD. Studies focused on delineating the ethnicity-specific factors and pathological role of *RNF213* variants in MMD and ICASO are needed.

## Additional files

Additional file 1: Figure S1. Sensitivity analysis of the association of *RNF213* p.R4810K with MMD and ICASO under a dominant model (TIFF 1024 kb)

Additional file 2: Table S1. Other rare variants of *RNF213* identified in different populations (XLSX 14 kb)

Additional file 3: Literature list (XLSX 67 kb)

## Abbreviations

ICASO: Non-moyamoya intracranial major artery stenosis/occlusion
MMD: Moyamoya disease
*RNF213*: The ring finger protein 213

## References

[1] Research Committee on the Pathology and Treatment of Spontaneous Occlusion of the Circle of Willis; Health Labour Sciences Research Grant for Research on Measures for Infractable Diseases (2012). Guidelines for diagnosis and treatment of moyamoya disease (spontaneous occlusion of the circle of Willis). Neurol Med Chir (Tokyo).

[2] Scott RM, Smith ER (2009). Moyamoya disease and moyamoya syndrome. N Engl J Med.

[3] Hayashi K, Horie N, Izumo T, Nagata I (2014). A nationwide survey on unilateral moyamoya disease in Japan. Clin Neurol Neurosurg.

[4] Ahn IM, Park DH, Hann HJ, Kim KH, Kim HJ, Ahn HS (2014). Incidence, prevalence, and survival of moyamoya disease in Korea: a nationwide, population-based study. Stroke.

[5] Starke RM, Crowley RW, Maltenfort M, Jabbour PM, Gonzalez LF, Tjoumakaris SI (2012). Moyamoya disorder in the United States. Neurosurgery.

[6] Miao W, Zhao PL, Zhang YS, Liu HY, Chang Y, Ma J (2010). Epidemiological and clinical features of moyamoya disease in Nanjing, China. Clin Neurol Neurosurg.

[7] Kim JS (2016). Moyamoya disease: epidemiology, clinical features, and diagnosis. J Stroke.

[8] Miyamoto S, Yoshimoto T, Hashimoto N, Okada Y, Tsuji I, Tominaga T (2014). Effects of extracranial-intracranial bypass for patients with hemorrhagic moyamoya disease: results of the Japan Adult Moyamoya Trial. Stroke.

[9] Yamauchi T, Tada M, Houkin K, Tanaka T, Nakamura Y, Kuroda S (2000). Linkage of familial moyamoya disease (spontaneous occlusion of the circle of Willis) to chromosome 17q25. Stroke.

[10] Kuroda S, Houkin K (2008). Moyamoya disease: current concepts and future perspectives. Lancet Neurol.

[11] Kamada F, Aoki Y, Narisawa A, Abe Y, Komatsuzaki S, Kikuchi A (2011). A genome-wide association study identifies RNF213 as the first moyamoya disease gene. J Hum Genet.

[12] Liu W, Morito D, Takashima S, Mineharu Y, Kobayashi H, Hitomi T (2011). Identification of RNF213 as a susceptibility gene for moyamoya disease and its possible role in vascular development. PLoS One.

[13] Wu Z, Jiang H, Zhang L, Xu X, Zhang X, Kang Z (2012). Molecular analysis of RNF213 gene for moyamoya disease in the Chinese Han population. PLoS One.

[14] Cecchi AC, Guo D, Ren Z, Flynn K, Santos-Cortez RL, Leal SM (2014). RNF213 rare variants in an ethnically diverse population with moyamoya disease. Stroke.

[15] Lee MJ, Chen YF, Fan PC, Wang KC, Wang K, Wang J (2015). Mutation genotypes of RNF213 gene from moyamoya patients in Taiwan. J Neurol Sci.

[16] Moteki Y, Onda H, Kasuya H, Yoneyama T, Okada Y, Hirota K, et al. Systematic validation of RNF213 coding variants in Japanese patients with moyamoya disease. J Am Heart Assoc. 2015;4 doi:10.1161/jaha.115.001862. 10.1161/JAHA.115.001862 PMC4599414 25964206

[17] Shoemaker LD, Clark MJ, Patwardhan A, Chandratillake G, Garcia S, Chen R (2016). Disease variant landscape of a large multiethnic population of moyamoya patients by exome sequencing. G3 (Bethesda).

[18] Huang Y, Cheng D, Zhang J, Zhao W (2016). Association between the rs112735431 polymorphism of the RNF213 gene and moyamoya disease: a case-control study and meta-analysis. J Clin Neurosci.

[19] Zhang Q, Liu Y, Zhang D, Wang R, Zhang Y, Wang S, et al. RNF213 as the major susceptibility gene for Chinese patients with moyamoya disease and its clinical relevance. J Neurosurg. 2016:1–8. doi:10.3171/2016.2.jns152173. 10.3171/2016.2.JNS152173 27128593

[20] Liu W, Senevirathna ST, Hitomi T, Kobayashi H, Roder C, Herzig R (2013). Genomewide association study identifies no major founder variant in Caucasian moyamoya disease. J Genet.

[21] Miyawaki S, Imai H, Takayanagi S, Mukasa A, Nakatomi H, Saito N (2012). Identification of a genetic variant common to moyamoya disease and intracranial major artery stenosis/occlusion. Stroke.

[22] Miyawaki S, Imai H, Shimizu M, Yagi S, Ono H, Mukasa A (2013). Genetic variant RNF213 c.14576G>A in various phenotypes of intracranial major artery stenosis/occlusion. Stroke.

[23] Bang OY, Ryoo S, Kim SJ, Yoon CH, Cha J, Yeon JY (2015). Adult moyamoya disease: a burden of intracranial stenosis in East Asians?. PLoS One.

[24] Bang OY, Chung JW, Cha J, Lee MJ, Yeon JY, Ki CS (2016). A polymorphism in RNF213 is a susceptibility gene for intracranial atherosclerosis. PLoS One.

[25] Kim YJ, Lee JK, Ahn SH, Kim BJ, Kang DW, Kim JS (2016). Nonatheroscleotic isolated middle cerebral artery disease may be early manifestation of moyamoya disease. Stroke.

[26] Liu W, Hitomi T, Kobayashi H, Harada KH, Koizumi A (2012). Distribution of moyamoya disease susceptibility polymorphism p.R4810K in RNF213 in East and Southeast Asian populations. Neurol Med Chir (Tokyo).

[27] Wang X, Zhang Z, Liu W, Xiong Y, Sun W, Huang X (2013). Impacts and interactions of PDGFRB, MMP-3, TIMP-2, and RNF213 polymorphisms on the risk of moyamoya disease in Han Chinese human subjects. Gene.

[28] Kobayashi H, Brozman M, Kyselova K, Viszlayova D, Morimoto T, Roubec M (2016). RNF213 rare variants in Slovakian and Czech moyamoya disease patients. PLoS One.

[29] Koizumi A, Kobayashi H, Hitomi T, Harada KH, Habu T, Youssefian S (2016). A new horizon of moyamoya disease and associated health risks explored through RNF213. Environ Health Prev Med.

[30] MacArthur DG, Manolio TA, Dimmock DP, Rehm HL, Shendure J, Abecasis GR (2014). Guidelines for investigating causality of sequence variants in human disease. Nature.

[31] Sun XS, Wen J, Li JX, Lai R, Wang YF, Liu HJ (2016). The association between the ring finger protein 213 (RNF213) polymorphisms and moyamoya disease susceptibility: a meta-analysis based on case-control studies. Mol Gen Genomics.

[32] Minelli C, Thompson JR, Abrams KR, Thakkinstian A, Attia J (2009). The quality of meta-analyses of genetic association studies: a review with recommendations. Am J Epidemiol.

[33] Little J, Higgins JP, Ioannidis JP, Moher D, Gagnon F, von Elm E (2009). Strengthening the reporting of genetic association studies (STREGA): an extension of the STROBE Statement. Hum Genet.

[34] Stang A (2010). Critical evaluation of the Newcastle-Ottawa scale for the assessment of the quality of nonrandomized studies in meta-analyses. Eur J Epidemiol.

[35] Miyatake S, Miyake N, Touho H, Nishimura-Tadaki A, Kondo Y, Okada I (2012). Homozygous c.14576G>A variant of RNF213 predicts early-onset and severe form of moyamoya disease. Neurology.

[36] Huang Y, Cheng D, Zhang J, Zhao W, Luo M (2015). Association between RNF213 gene polymorphisms and the genetic susceptibility of adult moyamoya disease of Zhuang population in Guangxi. J Apoplexy Nerv Dis.

[37] Kim EH, Yum MS, Ra YS, Park JB, Ahn JS, Kim GH (2016). Importance of RNF213 polymorphism on clinical features and long-term outcome in moyamoya disease. J Neurosurg.

[38] Park MG, Shin JH, Lee SW, Park HR, Park KP (2017). RNF213 rs112735431 polymorphism in intracranial artery steno-occlusive disease and moyamoya disease in Koreans. J Neurol Sci.

[39] Jang MA, Chung JW, Yeon JY, Kim JS, Hong SC, Bang OY (2017). Frequency and significance of rare RNF213 variants in patients with adult moyamoya disease. PLoS One.

[40] Shinya Y, Miyawaki S, Imai H, Hongo H, Ono H, Takenobu A, et al. Genetic analysis of ring finger protein 213 (RNF213) c.14576G>A in intracranial atherosclerosis of the anterior and posterior circulations. J Stroke Cerebrovasc Dis. 2017; doi:10.1016/j.jstrokecerebrovasdis.2017.06.043. 10.1016/j.jstrokecerebrovasdis.2017.06.043 28797616

[41] Shang D, Shi C, Mao C, Qin J, Gao Y, Zhao L (2015). Association between ring finger protein 213 gene polymorphism and ischemic stroke in a Chinese Han population. J Apoplexy Nerv Dis.

[42] Zhang T, Guo C, Liao X, Xia J, Wang X, Deng J, et al. Genetic analysis of RNF213 p.R4810K variant in non-moyamoya intracranial artery stenosis/occlusion disease in a Chinese population. Environ Health And Prev Med. 2017;22 doi:10.1186/s12199-017-0649-0. 10.1186/s12199-017-0649-0 PMC5664791 29165136

[43] Xue S, Cheng W, Wang W, Song H, Feng WW, Ovbiagele B. Genetic variant RNF213 in non-MMD intracranial major artery stenosis/occlusion in Chinese Han population and HR-MRI findings. Stroke. 2017;48(Suppl 1):AWP151.

[44] Mineharu Y, Takenaka K, Yamakawa H, Inoue K, Ikeda H, Kikuta KI (2006). Inheritance pattern of familial moyamoya disease: autosomal dominant mode and genomic imprinting. J Neurol Neurosurg Psychiatry.

[45] Sonobe S, Fujimura M, Niizuma K, Nishijima Y, Ito A, Shimizu H (2014). Temporal profile of the vascular anatomy evaluated by 9.4-T magnetic resonance angiography and histopathological analysis in mice lacking RNF213: a susceptibility gene for moyamoya disease. Brain Res.

[46] Hitomi T, Habu T, Kobayashi H, Okuda H, Harada KH, Osafune K (2013). Downregulation of Securin by the variant RNF213 R4810K (rs112735431, G>A) reduces angiogenic activity of induced pluripotent stem cell-derived vascular endothelial cells from moyamoya patients. Biochem Biophys Res Commun.

[47] Hitomi T, Habu T, Kobayashi H, Okuda H, Harada KH, Osafune K (2013). The moyamoya disease susceptibility variant RNF213 R4810K (rs112735431) induces genomic instability by mitotic abnormality. Biochem Biophys Res Commun.

[48] Kobayashi H, Yamazaki S, Takashima S, Liu W, Okuda H, Yan J (2013). Ablation of Rnf213 retards progression of diabetes in the Akita mouse. Biochem Biophys Res Commun.

[49] Kobayashi H, Matsuda Y, Hitomi T, Okuda H, Shioi H, Matsuda T, et al. Biochemical and functional characterization of RNF213 (Mysterin) R4810K, a susceptibility mutation of moyamoya disease, in angiogenesis in vitro and in vivo. J Am Heart Assoc. 2015;4 doi:10.1161/jaha.115.002146. 10.1161/JAHA.115.002146 PMC4608092 26126547

[50] Scholz B, Korn C, Wojtarowicz J, Mogler C, Augustin I, Boutros M (2016). Endothelial RSPO3 controls vascular stability and pruning through non-canonical WNT/Ca(2+)/NFAT signaling. Dev Cell.

[51] Banh RS, Iorio C, Marcotte R, Xu Y, Cojocari D, Rahman AA (2016). PTP1B controls non-mitochondrial oxygen consumption by regulating RNF213 to promote tumour survival during hypoxia. Nat Cell Biol.

