# Supplementary figures and images for: Rare variants of RNF213 and moyamoya/non-moyamoya intracranial artery stenosis/occlusion disease risk: a meta-analysis and systematic review
Source: Environ Health Prev Med. 2017 Nov 2;22:75. doi: 10.1186/s12199-017-0680-1 (PMC5667490; doi:10.1186/s12199-017-0680-1)

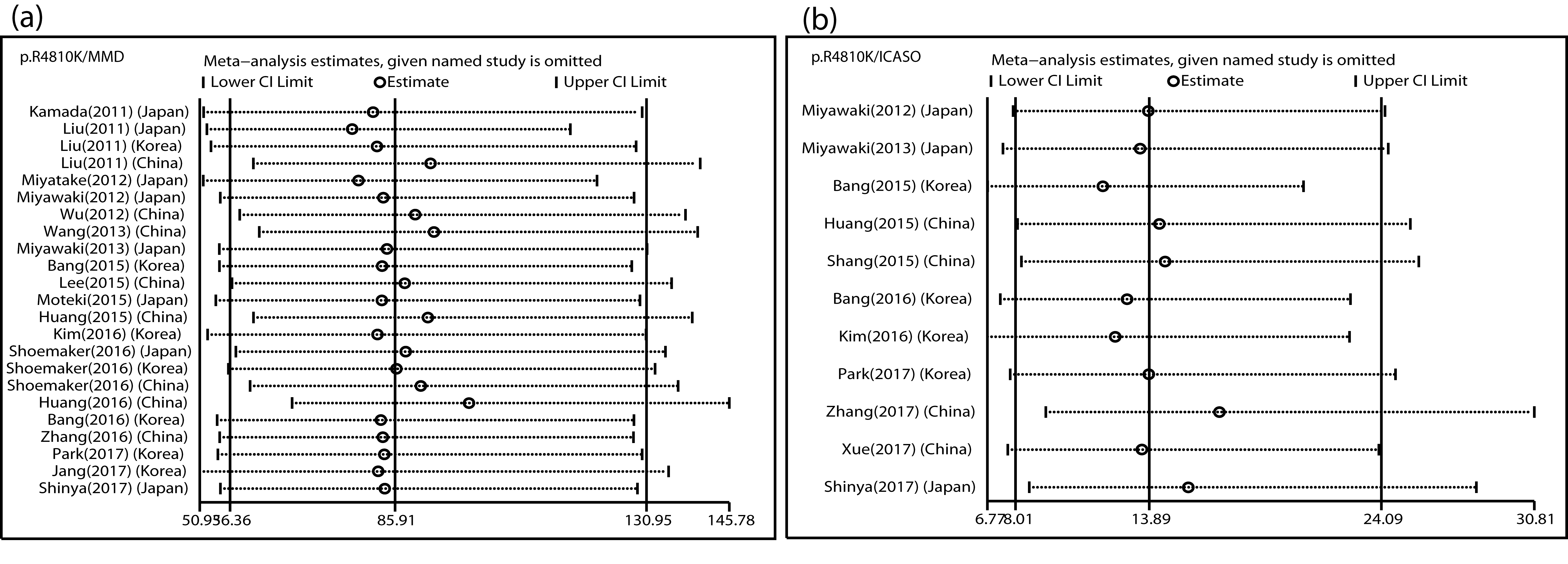

Supplement: Supplementary file 1 — Sensitivity analysis of the association of RNF213 p.R4810K with MMD and ICASO under a dominant model (TIFF 1024 kb) [file 12199_2017_680_MOESM1_ESM.tif]
